# Supplementary material for: Are carbon emissions trading and green financial instruments synergistic? -Comprehensive quantitative research based on content analysis
Source: PLoS One. 2024 Mar 7;19(3):e0298601. doi: 10.1371/journal.pone.0298601 (PMC10919601; doi:10.1371/journal.pone.0298601)
Supplement: S1 Data — (ZIP) [file pone.0298601.s001.zip › Data/地级市Z_F工作报告.zip!/─┌├╔╣┼/║⌠┬╫▒┤╢√╩╨/└┤╫╘╣1⁄2╓┌║┼í╛┬φ┐╦╩2╛▌═°í┐_2016.docx]

各位代表：

现在，我代表呼伦贝尔市人民政府向大会报告工作，请连同《呼伦贝尔市国民经济和社会发展第十三个五年规划纲要（草案）》一并审议，并请市政协委员和列席会议的同志提出意见。

一、“十二五”时期和2015年经济社会发展回顾

“十二五”时期，是我市发展进程中具有里程碑意义的时期。五年来，特别是党的十八大以来，面对复杂多变的经济形势和艰巨繁重的改革发展稳定任务，市政府在自治区党委、政府和市委的坚强领导下，团结带领全市各族干部群众，深入贯彻落实党中央的重大决策部署，深入贯彻落实习近平总书记系列重要讲话和考察内蒙古重要讲话精神，深入贯彻落实自治区“8337”发展思路，坚持“美丽发展、科学崛起、共享繁荣”总体要求，统筹稳增长、调结构、促改革、惠民生，推动经济社会发展水平上了一个新台阶，开创了全市经济社会发展的新局面。

综合经济实力迈上新台阶。2015年，地区生产总值完成1596亿元，五年增长0.7倍，年均增长10.8%，人均地区生产总值突破1万美元大关，这是我市经济社会发展的一个重要分水岭。50万元以上固定资产投资五年累计完成5661.4亿元，增长1.8倍，年均增长19.6%。公共财政预算收入完成103.3亿元，增长0.6倍，年均增长9.7%。规模以上工业总产值达到1287亿元，增长0.7倍，年均增长11.7%。社会消费品零售总额达到546亿元，增长0.9倍，年均增长13.1%。在宏观经济下行压力持续加大的情况下，实现了持续、稳定增长。

经济结构调整迈上新台阶。“五大基地”建设取得重要进展，三次产业结构由19.6：42.1：38.3优化为16.5:44.6:38.9。种植业结构调整成效显著，玉米种植面积稳定在1000万亩以上，粮食产量达到124.2亿斤，实现“十二连增”。草原畜牧业转型升级步伐加快，农区畜牧业发展加速，牧业年度牲畜存栏达到2136.7万头只，农区牲畜头数占比达到72.2%。农牧业产业化力度加大，农畜产品加工业实现销售收入425亿元，是2010年的2.7倍。农畜产品加工转化率达到50%，五年提高15.8个百分点。

工业结构优化升级。引进培育了阜丰集团、北方药业、呼伦贝尔肉业、云南驰宏等一批大型企业，玉米加工、生物制药、绿色农畜产品加工、有色金属等新的优势产业发展实现突破。非资源型产业占工业总产值比重达到67.5%，五年提高3.7个百分点，成为拉动工业增长的主要力量。

服务业蓬勃发展。旅游规模达到1417万人次，年均增长12.3%；旅游收入完成448.4亿元，年均增长27.9%。金融、保险等行业大幅增长，社会融资总规模连续三年超过存款总额，达到1587亿元。非公经济活跃度明显增强，占全市GDP比重达到65％，五年增加14.9个百分点。

生态环境保护迈上新台阶。生态建设五年投入63.4亿元，是“十一五”的3.6倍。造林619万亩，森林覆盖率达到51.4%，新增活立木蓄积量1.2亿立方米，我市荣获“国家森林城市”和“低碳试点城市”称号。累计发放草原生态补助奖励资金26亿元，禁牧和草畜平衡1.04亿亩，惠及873个嘎查村、13.7万户、39万人。沙区综合治理累计投入8.8亿元，完成541.9万亩，实现了荒漠化和沙化面积“双减少”。推进呼伦湖生态环境综合治理，水位上涨3.2米，水域面积达到2038平方公里。绿色矿山、和谐矿区建设持续推进。完成了自治区下达的节能减排节水任务。

城乡基础设施建设迈上新台阶。城乡建设成效显著。五年投入1664亿元，是“十一五”的3.9倍。房屋建设连续五年超千万平方米，海拉尔火车站、爱心家园、市政务服务中心、民生大厦、“三馆一湖”等一大批重点工程投入使用。累计实施供水、河道整治等市政公用基础设施项目1400多个，完成投资377.4亿元，是“十一五”的3.3倍，人均道路和公园绿地面积分别增长82%和53%，伊敏河综合治理工程主体完工。城市新区建设有序推进，中心城区建成区面积达到84.5平方公里。

基础设施建设全面提速。五年投入535.4亿元，是“十一五”的1.6倍。高速公路达到453公里，一级公路达到543公里，全市公路通车里程达到23483公里。铁路运营里程达到2236公里。海拉尔机场旅客吞吐量达到183万人次，扎兰屯支线机场和根河、阿荣旗、新右旗三个通用机场建成，机场群建设初具规模。红花尔基水利枢纽投入使用，扬旗山水利枢纽工程全面完工。供电能力明显改善，基本形成了以500千伏和220千伏为主网架、110千伏及以下为配网的电网架构。

改革开放迈上新台阶。坚持问题导向，全面深化改革。十八届三中全会以来，累计实施经济、生态、文化、社会领域改革234项，形成成果129项。政府机构改革如期完成，精简、取消、下放539项行政审批事项，占全部审批事项的80%。呼伦贝尔经济技术开发区升级为国家级开发区，组建了呼伦贝尔农垦集团、城投集团等9个投融资建设主体，为促进经济社会发展发挥了重要作用。三家非公企业在新三板挂牌，实现了直接上市融资零的突破。农村牧区综合改革稳步推进，土地、草牧场确权登记颁证试点工作进展顺利，流转土地1000万亩、草牧场1500万亩。

主动融入“一带一路”战略，扩大对外开放。呼伦贝尔中俄蒙合作先导区上升为国家战略，满洲里国家重点开发开放试验区建设加快推进，蒙古国驻呼伦贝尔领事馆正式开馆。“呼伦贝尔--天人合一的地方”城市宣传片亮相纽约时代广场，提升了呼伦贝尔的知名度和美誉度。对外贸易稳定增长，进出口总额累计完成137.8亿美元。招商引资成效显著，累计引进国内（市外）资金3496.2亿元，是“十一五”的2.1倍。

人民生活水平迈上新台阶。民生投入大幅增加。民生支出占公共财政预算支出62.5%，五年年均增长16%。城镇常住居民人均可支配收入达到26844元，五年增加11987元，年均增长11.4%；农村牧区常住居民人均可支配收入达到11632元，五年增加5337元，年均增长13.9%，城乡居民收入增速双双跑赢GDP和财政收入增速。“十个全覆盖”工程累计投入110.8亿元，1207个行政村85%实现全覆盖，279个自然村屯实现全覆盖，农村牧区面貌发生了根本性变化。扶贫开发成效明显，五年投入101.2亿元，脱贫21.2万人。保障性安居工程五年投入400亿元，建设、改造住房28万套，其中城市棚户区5.9万套，建立起了多层次、全覆盖的城镇住房保障体系。创业就业工程扎实推进，新增城镇就业18.2万人。累计发放购煤补助4.1亿元，23万多户低收入农牧民家庭受益。发放助学金1.9亿元，帮助5355名低保家庭的孩子圆了大学梦。为每个零就业家庭至少解决1人就业，实现了动态清零。

社会保障制度逐步健全。城乡居民基本养老保险制度实现全覆盖。城镇低保标准由每月282元提高到468元，农村牧区低保标准由每年1847元提高到3300元。蔬菜仓储设施达到20万立方米,建成75家蔬菜平价超市。投资15亿元，实施中心城区集中供热和“拆炉并网”工程，拆除锅炉120座，新增集中供热325.8万平方米，有效解决了城市燃煤污染问题，中心城区的天更蓝，空气更清新。

实施创新驱动战略，完成重大科技成果转化项目52项，科技创业服务中心投入使用，科技引领支撑作用进一步增强。教育五年投入25亿元，各级各类教育协调发展，教育质量明显提升。公共卫生服务能力和医疗服务水平持续增强，基层医疗卫生机构运行新机制基本建立，常住人口新农合参合率达到99%以上。计划生育工作扎实推进，人口出生水平保持稳定。公共文化服务设施全部免费开放，民族历史话剧《拓跋鲜卑》、广播剧《彩虹升起的地方》荣获国家“五个一工程奖”。全民健身设施条件明显改善，竞技体育水平大幅提高。食品药品监管实现全覆盖。安全生产形势保持平稳。档案史志、市场监管、气象、人防、地震、工青妇、红十字、科协、关工委、残联和老龄等工作都有新进展。

民主法制建设和政府自身建设迈上新台阶。全面推进依法行政、政务公开和科学民主决策，自觉接受人大依法监督和政协民主监督，认真听取各民主党派、工商联和无党派人士意见，累计办理人大代表议案5件，意见、批评和建议666件，政协委员提案1165件。建立了政府重大行政决策合法性审查制度和法律顾问制度，规范性文件的制定、备案、审查机制进一步完善。依法治市稳步推进，获得国家“六五”普法先进城市称号。严格执行中央八项规定、自治区28项具体要求和市委相关要求，行政监察和审计监督得到加强，“四风”问题得到有效遏制，“三公”经费缩减31.2%。

精神文明建设持续加强，成功承办了全国农村精神文明建设工作经验交流会。社会治安综合治理深入开展，平安呼伦贝尔建设成效明显。市本级累计化解信访积案4595件，全市信访总量下降27.8%。社区建设和社会管理成绩显著，我市被评为全国“社会管理综合治理优秀市”。军地关系更加和谐，双拥和边防工作得到加强。民族团结、社会稳定、边疆安宁的大好局面进一步巩固。

各位代表，2015年是我市经济社会发展进程中不平凡的一年。面对速度换挡、结构调整、动力转换的经济发展新常态，我们全面落实市委三届六次全委会议精神，按照“立足当前稳增长、着眼长远调结构、全面改革增活力、扎扎实实惠民生”的工作安排，奋力开拓，扎实工作，全面完成了市人大三届三次会议确定的主要目标任务：地区生产总值增长8.1%，公共财政预算收入增长7.6%，限额以上固定资产投资增长15.3%，城镇和农村牧区常住居民人均可支配收入分别增长8.3%和8.2%。全市经济社会发展实现了总体平稳、稳中有进的预期目标。一年来，我们主要做了以下工作。

（一）着力稳增长。把稳增长作为新常态下经济工作的首要任务。发挥投资对稳增长的关键作用。健全完善重点项目和重大事项专项推进机制。开复工重点项目172个，完成投资370亿元。实施了城建及公路、铁路、民航、水利、电网等一批重大项目，为稳增长发挥了重要作用。多措并举稳定企业生产。出台工业经济稳增长十条措施和扶持小微企业加快发展等一系列措施，落实国家结构性减税和取消降低收费等政策，推动电力直接交易及电力直供，降低企业运行成本近3亿元，帮助82户停产半停产企业恢复生产。发放援企稳岗资金5000万元，100多家企业受到资助，惠及企业员工11万人。金融创新拓宽了融资渠道。金融机构新增贷款187.5亿元，存贷比72.6%，提高9.2个百分点。积极推广政府和社会资本合作模式，31个PPP项目列入自治区和国家项目库，完成投资23.8亿元。

（二）着力调结构。大力发展现代农牧业。粮食增产1.1亿斤，牲畜增加181.3万头只，粮食总产、牲畜存栏均创历史新高。新增高效节水灌溉面积56.3万亩，科技对农牧业贡献率达到55%，无公害、绿色、有机农畜产品认证和农畜产品地理标志认证达到304个，农牧民合作社达到7786家，畜牧业产值占一产比重达到35%。推动工业转型升级。加快传统优势产业技术改造，延伸链条，推进新兴产业做大规模，绿色农畜产品加工、有色金属、制药产业和玉米深加工等产业产值分别达到342.4亿元、98.6亿元、73.9亿元和146.8亿元，分别增长6.6%、46.6%、47.6%和29.1%，成为工业经济新的增长点。现代服务业持续发展。把旅游业作为战略性支柱产业来培育，带动了第三产业繁荣发展，第三产业增加值完成621.5亿元，增长9.3%。加快发展电子商务，跨境电子商务产业园入驻电商企业39家。

（三）着力促改革。全面简政放权。完成了“三单”编制，向社会公布权力清单6764项、责任清单237项、负面清单35项。市政务服务中心和公共资源交易中心投入运行，实现了部门、事项、收费100%进驻，政府采购、国有产权交易、工程建设招投标、土地使用权和矿权交易等公共资源集中进场交易。推行“先照后证”制度，完成了“三证合一”工作，新增市场主体28127户、注册资本金173.8亿元。加强生态文明制度建设。推动国有重点林区、地方国有林场改革，全面停止天然林商业性采伐。启动了国家自然资源资产负债表编制试点和领导干部自然资源资产离任审计试点工作。构建开放型经济新体制。满洲里综合保税区和中俄蒙合作先导区建设规划获得国家批复。满洲里在全国率先启动卢布现钞使用试点。额布都格、阿日哈沙特口岸实现临时常年开放。

（四）着力惠民生。举全市之力推进“十个全覆盖”工程。全年完成投资95亿元，改造危房90963户，街巷硬化3166公里，修建院墙652.3万延长米、甬道116.5万延长米，植树500余万株。扎实推进扶贫开发、百姓安居和创业就业工程。3.5万农牧民摆脱了贫困，3.5万城镇居民喜迁新居，城镇新增就业2.9万人，创业园和孵化基地达到74家。着力提高社会保障水平。企业养老金月人均增加193元，城镇居民医保财政补助标准年提高60元。加快发展各项社会事业。启动了种子工程。组建了岭西和岭东两个职教集团，扎兰屯职业学院开始招生。蒙古族源与元朝帝陵综合考察研究项目科研中心开工建设。旗县级公立医院改革全面推开。足球改革进展顺利。大力促进社会和谐。启动了争创全国文明城各项准备工作。各类安全事故起数下降12%。严厉打击各类违法犯罪活动，群众安全感明显增强。扎实开展“三严三实”专题教育，政府作风建设常态化机制正在形成。

（五）着力保生态。认真贯彻落实新《环境保护法》。集中开展大气、水污染防治和矿区环境综合整治等环保专项行动，自治区及市本级92项减排任务全面完成。推进呼伦湖流域综合治理。开展了呼伦湖环境整治“百日攻坚”行动，排查整治48家沿湖周边经营户和企业，全流域生态环境得到改善。继续推进天保二期、草原生态补奖、沙区综合治理等生态工程。造林绿化120万亩，草畜平衡7508万亩、禁牧2850万亩，完成沙区综合治理100万亩。

各位代表，经过五年的艰辛努力，我们全面完成了“十二五”的主要目标任务，成绩来之不易，呼伦贝尔已经站在了一个新的历史起点上。这些成绩的取得是自治区党委、政府和市委坚强领导的结果，是市人大、政协有效监督和大力支持的结果，是全市各族人民团结奋斗的结果。在这里，我代表市人民政府，向全市各族人民，向为呼伦贝尔建设和发展作出贡献的同志们，向驻呼军警部队官兵，向人大代表、政协委员，向所有关心、支持和帮助呼伦贝尔发展的朋友们，表示衷心的感谢和崇高的敬意!

在肯定成绩的同时，我们也清醒地认识到存在的困难、矛盾和挑战：我市是欠发达的边疆民族地区，经济总量不大，综合实力不强，城乡、区域、经济社会发展不协调，基础设施和基本公共服务相对落后，城乡居民收入水平不高，与全国全区同步建成小康社会的任务艰巨繁重。我市属于资源型地区，产业结构单一，原字号、低端化特征明显，非资源型产业、战略性新兴产业和现代服务业发展滞后，推进传统产业新型化、新兴产业规模化、支柱产业多元化，构建多元发展、多极支撑现代产业体系的任务艰巨繁重。我市生态环境比较脆弱，敏感度和社会关注度高，落实主体功能区规划，守住生态红线，推进绿色转型，实现美丽与发展双赢，打造祖国北疆生态安全屏障的任务艰巨繁重。我市市场发育不充分，发展环境不优，民营经济发展不足，科技支撑薄弱，人才、资本短缺，创新发展的能力和活力不强，新常态下转变发展方式、调整经济结构、实现要素驱动向创新驱动转变的任务艰巨繁重。我市地处偏远，远离大城市和中心市场，处于铁路、公路、航空、电网、信息等各种通道的神经末端，难以承接大城市、大市场的带动和辐射，生产、生活、物流成本相对较高，加快基础设施建设，补齐发展“短板”的任务艰巨繁重。还要看到，随着财税改革深入推进，主体税种增长放缓，政策性减税因素增多，新的增长点培育尚待时日，民生支出刚性增长，财政收支矛盾加剧，保民生、保发展的任务艰巨繁重。各级政府自身改革正在进行中，职能转变尚在路上，部分干部存在“为官不为”问题，能力不足“不能为”、动力不足“不想为”和担当不足“不敢为”等现象不同程度存在，打造风清气正、干事创业发展环境的任务艰巨繁重。我们要坚持问题导向和底线思维，切实应对和解决好这些困难、矛盾和挑战。

二、“十三五”时期经济社会发展的主要目标和任务

“十三五”时期，是我市全面建成小康社会的决胜期，是全面深化改革的攻坚期，是全面推进依法治市的关键期。根据党的十八届五中全会、自治区党委九届十四次全委会精神，市委三届七次全委会议确定了“十三五”时期我市经济社会发展的总体要求、主要预期目标和重点任务，已经全面体现在《呼伦贝尔市国民经济和社会发展第十三个五年规划纲要（草案）》中，提交本次大会审议。

今后五年，要坚持发展是第一要务，推动经济持续健康发展，全面建成小康社会。以国家实施供给侧结构性改革为发力点，发挥投资的关键性作用，规划实施重点项目2600个，全社会固定资产投资年均增长10%以上、累计达到6200亿元以上。挖掘内需潜力，培育消费热点，优化消费环境，发挥消费的基础性作用。努力保持经济中高速增长，地区生产总值年均增长7.5%，公共财政预算收入年均增长6%，与全国全区同步全面建成小康社会。

今后五年，要坚持向改革要动力，着力提高发展质量和效益，实现创新发展。进一步理顺政府和市场的关系，培育形成一批有活力、能创新、善协作、带动就业强的市场主体。加快传统产业和重点领域创新，发展“互联网+”经济，推动三次产业与信息化深度融合，战略性新兴产业增加值占地区生产总值比重明显提高，实现产业向中高端迈进。大力推进大众创业、万众创新，提升高校的创新能力，完善现代职业教育体系，造就各类创新型应用人才，打造一批不同规模、不同类型的创业基地和创业平台。重点领域和关键环节改革取得决定性成果，着力破解制约经济社会发展的体制机制障碍，激发发展新活力，再造发展新优势。

今后五年，要坚持全力以赴调结构、转方式，着力增强发展的整体性，实现协调发展。按照“传统产业新型化、新兴产业规模化、支柱产业多元化”的要求协调三次产业发展。农牧业现代化水平明显提高，工业转型升级实现突破，金融、旅游、物流等现代服务业的支撑作用明显提升，科技对经济增长的贡献率逐步提高，社会消费品零售总额年均增长10%，多极发展、多元支撑的现代产业体系基本确立，三次产业结构调整为15∶40.5∶44.5。优化生产力空间布局，促进区域分工协作，大力发展县域经济，统筹推动岭东、岭上、岭西地区协调发展。推进城乡一体化发展，加快以人为核心的新型城镇化，打造生态宜居美丽宜游的草原名城，常住人口城镇化率达到72%。加强基础设施建设，构建综合交通运输体系，到2020年，全市公路通车里程达到28000公里以上，铁路网总长度达到2900公里以上，市域内一小时航空圈全面建成。加强水利基础设施建设，水利灌溉面积达到800万亩以上。电网和信息基础设施建设取得新突破。

今后五年，要坚持一以贯之推进生态建设，实现绿色发展。把生态环境保护作为全市发展的基本前提和首要任务，树立尊重自然、顺应自然、保护自然的理念，坚守生态红线，基本形成主体功能区布局，生态文明制度基本健全。资源开发利用效率大幅提高，能源和水资源消耗、建设用地、碳排放总量有效控制，完成节能减排等各项约束性指标。到2020年，森林面积达到1300万公顷以上，新增活立木蓄积量1.2亿立方米以上，草原植被盖度达到74%。森林、草原、河湖、湿地等生态系统得到有效保护与修复，祖国北疆生态安全屏障更加巩固。

今后五年，要坚持走出去引进来相结合，深度融入国家“一带一路”战略，实现开放发展。全面参与中俄蒙经济走廊建设，建成中俄蒙合作先导区，满洲里国家重点开发开放试验区功能全面发挥，与俄蒙互联互通的基础设施、跨境经济合作区等产业合作平台基本建成，经贸、投资、人文等多元合作机制全面建立，逐步实现由口岸门户通道向进出口加工基地和多元合作平台的转变，欧亚大陆桥枢纽地位更加巩固，对外开放的活力和动力充分释放。年货运综合能力达到1亿吨，旅客通关能力达到1500万人次，对东北、环渤海、长三角、珠三角等地区产业吸引能力不断提升，开放对全市经济发展的牵动力显著增强。

今后五年，要坚持民生为本，着力增进人民福祉，实现共享发展。按照“坚守底线、突出重点、完善制度、引导预期”的原则，共享发展成果，城镇和农村牧区常住居民人均可支配收入年均增速均达到9%，人均收入达到全国平均水平。坚决打赢脱贫攻坚战，到2017年，4个自治区重点贫困县全部摘帽，到2020年，2个国家重点贫困县全部摘帽，全市6.27万贫困人口全部实现稳定脱贫。“十个全覆盖”、城镇棚户区改造等重大民生工程全面完成。全力承办好第十四届全国冬季运动会和内蒙古自治区第九届少数民族传统体育运动会。就业、教育、文化、社保、医疗、住房等公共服务体系和社会保障体系更加健全，基本公共服务均等化水平稳步提高。

今后五年，要推进和谐呼伦贝尔和法治呼伦贝尔建设，筑牢祖国北疆安全稳定屏障。巩固发展平等团结互助和谐的社会主义民族关系，少数民族集聚区、边境地区和谐发展，成为睦邻友好、民族和谐的示范区。全面推进依法治市，基本建成法治政府。加强和创新社会治理，加快构建全民共建共享的社会治理格局，建设平安幸福和谐呼伦贝尔。

三、2016年主要工作

2016年是实施“十三五”规划的开局之年，是推进结构性改革的攻坚之年,也是全面建成小康社会决胜阶段的第一年。今年政府工作的总体要求是：深入贯彻党的十八大、十八届三中、四中、五中全会、习近平总书记系列重要讲话和考察内蒙古重要讲话精神，全面贯彻自治区九届十四次全委会议和全区经济工作会议精神，认真落实市委三届七次全委会议和全市经济工作会议的工作部署，按照“五位一体”总体布局和“四个全面”战略布局，牢固树立和贯彻落实创新、协调、绿色、开放、共享的发展理念，适应经济发展新常态，坚持稳中求进工作总基调，坚持稳增长、调结构、惠民生、防风险，落实宏观政策要稳、产业政策要准、微观政策要活、改革政策要实、社会政策要托底的总体思路，战略上坚持持久战，战术上打好歼灭战，着力保持经济运行在合理区间，着力加强结构性改革，着力推进全面深化改革和扩大开放，着力加强生态环保，着力保障和改善民生，努力实现“十三五”时期全市经济社会发展的良好开局，以优异成绩迎接自治区成立70周年。

主要预期目标是：地区生产总值增长7.5%左右，限额以上固定资产投资增长13%，社会消费品零售总额增长10%，公共财政预算收入增长6.5%，城镇和农村牧区常住居民人均可支配收入均增长9%，规模以上工业增加值增长9%，主要约束性指标达到自治区要求。

做好2016年工作，必须认清形势、明确方向、统筹安排、精准施策。着重把握三个原则：一是适应新常态。核心是解决好速度换挡、结构优化和动力转换的问题，关键是把调结构、转方式贯穿于全年经济工作始终。二是贯彻新理念。必须坚定自觉地贯彻好“创新、协调、绿色、开放、共享”五大发展理念，对新发展理念细化、实化、政策化、机制化，使之成为发展的“指挥棒”和“红绿灯”。三是寻找新动能。关键是坚持改革，大胆创新，激发经济社会发展动力和活力，把创业创新作为内生发展动力的主要生成点，把经济增长的巨大潜力转变为现实，引领我市经济保持中高速增长。

为实现上述目标，重点做好八项工作：

（一）加强供给侧结构性改革，确保经济稳定增长。稳增长是今年工作的首要任务，要抓住供给侧结构性改革的契机，牢牢掌握稳增长的主动权。

准确把握中央政策。全面落实供给侧结构性改革五大任务，更加强调质量和效益，把政策范围内支持的事项作为工作的优先方向，在政策中找机遇、找项目，积极争取更多政策和资金，推动经济社会发展。加快推进齐齐哈尔-海拉尔-满洲里铁路客运专线、城市综合走廊、电力外送等一批重大项目、重大事项和重大政策的前期工作，尽快形成新的增长点。健全完善重大项目、重大事项和重大政策的推进机制。

提高投资的有效性和精准性。全年安排限额以上固定资产投资1044亿元，其中，第一产业投资100亿元，第二产业投资330亿元，第三产业投资614亿元。亿元以上重点项目218个，年度投资474亿元。重点投向绿色农畜产品加工、有色金属产业、生物制药、玉米深加工、机械制造、旅游、现代物流、城乡基础设施建设、公共服务和“十个全覆盖”等。

创新融资方式。创新政府投资方式，加大政府性担保金投入。充分发挥融资平台作用，做好产业发展基金、城市发展基金等基金设立发起工作。鼓励金融产品创新，综合运用信托、资产证券化等方式，扩大有效信贷投放，支持股权、债券融资。鼓励社会融资创新，发挥民间借贷服务中心平台作用，引导社会资本支持实体经济发展。推动企业上市融资。

去产能、去库存务求突破。正确处理巩固发展优势特色产业与化解过剩产能的关系，发挥企业主体作用，完善政府扶持政策。针对煤电产业产能相对过剩问题，推动上游煤电企业就地转化和兼并重组，形成下游产业优势。加快伊敏-兴安-乌兰浩特-科尔沁、海拉尔-牙克石-扎兰屯两条500千伏输电通道建设和大用户直供步伐，切实解决煤、电产业去库存问题。

降低企业成本。因业施策、因企施策，在简政放权、减税降费、金融支持和打造优质服务环境等方面出台具体政策，打好“组合拳”。继续开展涉企收费管理，开展大用户直供，加快园区重点企业铁路专用线等生产性物流配套建设，降低企业成本，增强企业竞争能力和发展后劲。

支持创业创新。营造创业创新环境、激发调动全社会创业创新激情。用信息化武装三次产业、实体经济。深入推进重点创新工程，完善优惠政策，强化市创业园示范引领作用，建设新的创业创新基地和呼伦贝尔经济技术开发区创业园，鼓励支持高校师生创业创新。继续加大非公企业发展政策措施支持力度，加强企业家队伍建设。鼓励引导企业运用互联网思维和信息技术谋划产业发展，发挥“互联网+”的乘数效应，推动网络经济快速发展。

（二）着力调整经济结构，转变发展方式。经济下行，从根本上讲是结构问题。调整经济结构，转变发展方式必须寻求新突破。

推动传统工业转型升级。支持企业通过技术改造、设备更新和信息化融合，提升加工转化能力和精深加工水平，形成产业综合成本优势。支持企业联合重组，加快褐煤多联产项目落地，推动煤电联营、煤化联营、煤冶联营，打造产业上下游要素链条，提高就地加工转化率，增强抵御市场风险能力。加大对能源资源消耗高、资源利用率低、污染减排压力大的企业的倒逼转型。扶优扶强，推动木材加工企业整合，引导木材加工产业向精深加工发展。优化岭东、岭西乳业布局，加快市域乳业企业兼并重组。

加快培育新的工业经济增长点。加快发展绿色食品加工业，制定三年发展规划，出台支持乳、肉、薯、草、油脂等加工行业的扶持措施。开工建设呼伦贝尔经济技术开发区海拉尔食品加工产业园。把有色金属产业作为主导产业培育，实施“1553”工程，推动有色金属探采选冶加全产业链发展，抓好国森矿业日采选5000吨铅锌多金属矿石项目，争取鄂伦春八岔沟日采选3000吨铅锌项目早日达产，加快新右旗哈拉胜铅锌矿“探转采”步伐，力争中国黄金集团10万吨铜冶炼项目开工。做优做大玉米加工产业，支持阜丰集团与世界高端企业合作，推动20万吨谷氨酸钠、鸿洋药业、北方药业三期等项目建设，全年加工转化玉米300万吨。打造牧业机械制造产业园，推动华德、瑞丰、蒙拓、沐禾等企业发展牧业机械和节水灌溉设备等装备制造业。完成呼伦贝尔经济技术开发区20万吨啤酒技术改造。加强大数据、云计算、物联网、互联网等新业态培育，推进中网科技云计算基地建设。

加快发展现代服务业。把现代服务业作为结构调整的主攻方向。把旅游业作为战略性支柱产业培育，全面启动国家级旅游业改革创新先行区工作。智慧旅游实现突破，扩大媒体和网络平台旅游宣传覆盖面，达到互联网全方位可查询水平。加快蒙古之源·蒙兀室韦历史文化园、两河圣山景区、拓跋鲜卑民族文化园、呼伦贝尔自然博物馆等项目建设。推动呼伦贝尔草原国家公园、满洲里中俄边境旅游区、柴河-阿尔山等5A级景区创建工作。规范旅游市场秩序，加强旅游执法，提升管理与服务水平。借助央视猴年春晚分会场宣传效应，推动旅游业再上新台阶，全年旅游接待人数和总收入保持两位数增长。加速发展物流业，以滨洲线节点城市为重点，突出抓好中俄蒙国际物流业，推动海拉尔、满洲里、牙克石、扎兰屯国际物流带建设。加快新业态、新产品发展，注重发展绿色、时尚、品质消费，鼓励社会资金投资养老机构、医疗健康、文体休闲、旅游地产等产业。推动金融、保险、电商、快递配送和环保产业等新型业态发展。

推动区域协调发展。制定区域发展规划，出台区域协调发展政策。加快岭东旗市发展，充分发挥农畜产品富集优势，以农牧业产业化带动区域经济加快发展，加快阿荣旗肉业、大杨树农产品加工基地建设，打造岭东新的经济增长极。岭西旗市要巩固提升传统煤电化产业，提高产业层次。岭上旗市要加快培育旅游、特色种养等接续替代产业，实现经济转型。口岸沿线旗市要依托中俄蒙合作先导区和满洲里国家重点开发开放试验区平台，以开放促发展。

（三）把发展现代农牧业作为结构调整的优先选择。按照农业供给侧结构性改革要求，大力推进农牧业现代化，着力强化物质装备和技术支撑，着力构建现代农牧业产业体系、生产体系、经营体系，推动粮经饲统筹、农林牧渔结合、种养加一体、一二三产业融合发展。全年粮食产量稳定在120亿斤以上，牲畜存栏稳定在2100万头只以上，肉类、牛奶产量稳定在25万吨和120万吨以上。

加快推动农牧业转型升级。优化农牧业生产结构和区域布局。继续推动草原畜牧业转型升级、农区畜牧业加快发展，提高畜牧业在大农业中的比重，实现畜牧业产值占一产比重达到37%。优化粮、经、草三元结构，提高经济作物和饲草饲料的种植比重，发展特色经济。

提升农业保障支撑能力。加快补齐农业短板，新增节水灌溉50万亩。新增高标准基本农田土地整治项目65万亩。强化现代农牧业科技创新推广体系建设，实施好鄂温克旗、阿荣旗国家级现代农业示范园区建设，加大基层科技服务力度，确保科技贡献率达到56%以上，农牧业机械化综合水平达到85%以上。

大力发展农牧业产业化。充分发挥呼伦贝尔农垦集团、肉业集团等龙头企业的带动作用，加快企业联合重组和技术改造步伐，确保农畜产品加工转化率达到53%。鼓励龙头企业自建基地，强力推进人畜分离,大力发展标准化、规模化养殖，全年新建和改扩建规模养殖场132个。推动龙头企业与农牧户、合作社建立利益联结机制，确保比例提高到48%。建设农畜产品绿色食品区域性大市场，继续推进“呼伦贝尔牛肉、羊肉、马铃薯、黑木耳”等品牌建设和农畜产品可追溯体系建设。发展互联网+现代农牧业，推动农牧业全产业链改造升级。

（四）统筹推进城乡建设，促进城乡协调发展。以补齐短板、强化配套，提升建设、管理和服务水平为导向，推进以人为核心的新型城镇化建设。全年投资280亿元，房屋建设面积保持在1000万平方米以上。

加强城乡规划设计。完善城镇总体规划和乡村规划，准确把握功能、产业特点和发展潜力，注重体现边城特色、林区特色、民族特色。编制《呼伦贝尔市中心城区一体化发展规划》。强化规划的刚性和权威性，做到一张蓝图绘到底，一届接着一届干。

提升城市综合承载能力。按照统一规划、适度超前、配套衔接的原则，大力实施城市基础设施和公共服务设施建设，全年投资70亿元以上。启动中心城区第二水源地建设，推进“美丽村镇”建设，实施农村牧区人居环境综合整治工程，提升城镇园林绿化水平，加快智慧城市建设，优化城市交通，启动城管执法体制改革，提高物业管理和服务质量。

加快新城建设和旧城改造。优先发展市中心城区，坚持市区共建、市区一体，完成伊敏河综合治理工程和“六二六小河”环境综合整治工程，加快二中新校区和市医院新区分院建设。统筹推动满洲里、牙克石、阿荣旗、扎兰屯等地新区建设。加快旧城区改造步伐,做好既有建筑节能改造、“城中村”改造、城市棚户区改造等项目，继续实施城区供热、供水老旧管网改造，确保设施完备、环境优美、宜居宜业。

持续推进基础设施建设。公路投资100亿元，重点建设海拉尔-满洲里、扎兰屯-阿荣旗高速公路，国道海拉尔-哈达图-拉布达林，省道满洲里-阿拉坦额莫勒等项目。争取开工尼尔基-大杨树、阿里河-库布春等公路，统筹谋划、加快推进林区公路建设，配合“十个全覆盖”全力推进通村通乡油路建设。铁路投资38亿元，加快滨洲铁路电气化改造、阿荣旗-莫旗铁路、呼伦贝尔经济技术开发区铁路专用线等项目建设，开通两伊铁路客运，加快齐齐哈尔-海拉尔-满洲里客运专线前期工作。民航投资3.5亿元，扎兰屯支线机场、新右旗、阿荣旗、莫旗通用机场实现通航，海拉尔机场扩建主体完成、新机场确定选址。水利投资25亿元，加快扎敦水库、尼尔基水库下游30万亩灌区建设，做好毕拉河水利枢纽、晓奇子水库和甘河大型扬水灌区前期工作, 争取Z866项目获得国家审批。电网投资30亿元，加快城网、农网改造步伐，加快已开工项目建设。推进呼伦贝尔-华北±800千伏电力外送通道前期工作。

（五）深化改革扩大开放，激发经济社会发展动力与活力。加大力度推动要素有序流动、资源高效配置、产业转型升级、市场深度融合，打造发展新优势。

全面深化改革。承接好国家和自治区2016年改革任务，坚持问题导向，推动全市各项改革落地生根。抓好政府自身改革，加大简政放权力度，再精简、下放一批审批事项，抓好“三单”目录贯彻执行，提高行政审批中心和公共资源交易平台办事效率和质量。推动财税体制改革。抓好主体功能区试点，积极争取国家政策支持。抓好农村牧区改革，加快推进土地、草牧场确权登记工作，推动土地、草牧场承包经营权流转、入股、信贷抵押等制度创新。加快培育新型农牧业经营主体，发展多种形式农牧业适度规模经营。巩固提升商事制度改革成果，推进电子营业执照和企业注册全程电子化，开展企业名称登记和企业简易注销改革工作，畅通市场主体退出机制。推动国有企业改革，积极支持森工集团改革，深化农垦集团改革，加快推进地方国有林场改革，完善企业法人治理结构。探索发展混合所有制经济，鼓励民间资本参与国有企业改革。

全面扩大开放。抓住国家实施“一带一路”和建设中蒙俄经济走廊重大机遇，突出沿边开放和沿线发展。推动中俄蒙合作先导区建设，争取自治区出台支持先导区建设的实施意见,尽快把国家政策细化、实化、具体化，确保各项政策落到实处。加快满洲里国家重点开发开放试验区建设，完善综合保税区功能，促进国际货运班列常态化运行。加强跨境合作，推动额布都格跨境经济合作区获批，启动项目招商，完成中俄蒙跨境合作区规划并启动报批。抓好跨境旅游，推动满洲里、额尔古纳设立跨境旅游合作区。加快口岸基础设施和互联互通建设，推进海拉尔-拉布达林-黑山头、室韦-莫尔道嘎铁路等项目前期工作，争取阿日哈沙特-乔巴山等公路早日开工，启动额布都格和室韦两座口岸大桥前期工作，打造中俄蒙跨境运输通道。发展进出口加工产业，抓好满洲里油菜籽、新左旗饲草、新右旗肉马等进口加工项目和诺永达拉果铅锌矿等境外资源加工项目。以海拉尔、牙克石、扎兰屯、大杨树等区域为重点，加快建设物流保税仓库，与满洲里保税区互为配合、互动发展。做好精准招商，积极承接东北、环渤海、长三角和珠三角等发达地区产业转移，全年引进国内（市外）资金560亿元。

（六）坚持绿色发展，推进生态文明建设。高度重视生态环境保护与治理，全力打造祖国北疆生态安全屏障。

坚守生态红线。坚持红线意识和底线思维，强化“绿水青山就是金山银山”的意识，全面落实国家环境保护制度和主体功能区规划，制定并严格执行森林、湿地、草原、沙区植被“四条红线”，坚决做到不符合主体功能区规划的项目不上、不符合环保要求的项目不上、不符合产业政策的项目不上。

加强生态环境保护和修复。继续加大草原、森林、河湖、湿地的保护与建设力度，实施国土绿化，天保二期，三北防护林，呼伦湖综合治理，新一轮草原生态补奖，公益林生态效益补偿、绿色矿山、和谐矿区等重点生态工程。全年造林绿化60万亩，沙地维护补建250万亩，退牧还草190万亩，禁牧和草畜平衡保持在1亿亩左右。

深化污染防治。落实最严格水资源管理制度，推进水生态文明试点城市建设。实施工业污染源全面达标排放计划，推进燃煤电厂超低排放，加强黑羊站水源地保护，做好污水处理厂及配套管网等工程建设。

推动低碳循环发展。探索发展绿色产业、环保产业，抓好12.5万亩红花尔基退化土地碳汇造林项目。

加大责任追究力度。全力做好自然资源资产负债表编制试点和领导干部自然资源资产离任审计试点工作。坚决打击毁林毁草开荒和超标准污染物排放等破坏生态环境的行为。坚决查处非法占用草场，与民争利等问题。

（七）加强民生和社会事业发展，维护社会和谐稳定。继续加大公共财政支出向民生和社会事业倾斜力度，全面落实各项惠民政策。集中力量推进“十个全覆盖”工程，继续强化组织领导，严格规划引领，多方筹措资金，保证工程质量。全年投资102.9亿元，确保5月底前全面完成1207个行政村建设任务，年底前全面完成1251个较大自然村屯建设任务，剩余的477个较小自然村屯开工危房改造和安全饮水工程，解决群众出行难问题。启动500个自然村屯的搬迁工作。全力以赴打好脱贫攻坚战，按照“六个精准”和“五个一批”的要求，深入实施“三到村三到户”、金融扶贫和“金种子”扶贫等工程，逐村逐户确定致富产业和帮扶措施，精准推进各类扶贫项目，确保2.5万人稳定脱贫。统筹抓好各项民生工程。抓好“百姓安居工程”，投资100亿元,开工各类保障性安居工程7.2万套。推进“创业就业工程”，实现全年城镇新增就业2.9万人，解决好高校毕业生和退役军人等重点人群的就业问题。继续实施“人才强市工程”，加大各类紧缺实用人才的培育和引进力度。提高社会保障能力，完善临时救助和大病救助制度，适度提高企业退休人员养老金、城乡低保、“五保”供养标准。继续推进“菜篮子”工程，加强反季节蔬菜和设施蔬菜基地建设，新建大棚2000座、温室360座。

推进科技、教育、卫生等各项事业发展。发挥科技企业孵化器培育和扶持科技型中小企业的功能，完善区域创新体系建设，促进科技成果转化。大力发展普惠性学前教育，促进义务教育均衡发展，加快普及高中阶段教育，努力办好特殊教育，全面提升民族教育水平，加快教育信息化“三通两平台”建设与应用，深化产教融合，促进校企合作，加快构建现代教育体系，支持呼伦贝尔学院向应用型本科院校转型发展。推动蒙古族源与元朝帝陵综合研究，推进市新闻中心、蒙古族源博物馆等项目建设。落实政府办医职责，巩固县级公立医院综合改革成果，提升基层医疗卫生服务能力，探索建立分级诊疗制度，稳妥实施全面两孩政策。完成食品检测中心建设，推进食品安全监管体系和应急处置能力建设，保障人民群众饮食用药安全。推进冰雪、足球、民族体育发展和城市居民10分钟健身圈建设，提高竞技体育水平和全市人民的健康水平。启动第十四届全国冬季运动会和内蒙古自治区第九届少数民族传统运动会筹备工作。支持红十字事业和公益慈善事业发展。加强统计、行政监察和财政财务审计工作。统筹推进市场监管、气象、人防、地震、档案史志等工作。

维护社会和谐稳定。深入推进平安呼伦贝尔建设，健全社会治安综合防控体系，防范和打击各类违法犯罪活动。加强社区建设，提高社会服务管理水平。压实信访工作责任，切实维护好群众合法权益。大力推进应急管理能力建设，严格落实安全生产三项责任，确保人民群众生命财产安全。推进国防动员和军警民边防体系建设，强化信息化管边控边各项措施，提升边境综合防卫管控能力。

（八）加强民主法治建设，打造人民满意政府。严格遵守党的政治纪律和政治规矩，增强看齐意识，坚定不移向党中央看齐，向党的理论和路线方针政策看齐。坚持依法治市，坚决维护宪法法律权威、人民权益和社会公平正义。做好“七五”普法工作，促进严格执法、公正司法、全民守法。

自觉接受人大及其常委会的法律监督、工作监督和人民政协的民主监督，配合人大做好地方性法规立法工作。主动听取人大代表、政协委员、民主党派、工商联、无党派人士及各人民团体的意见，认真办理人大代表议案、建议和政协委员提案。支持工青妇等群团组织工作。

落实党的民族宗教政策，大力扶持少数民族各项事业，切实增进“四个认同”，巩固发展平等团结互助和谐的社会主义民族关系，做好“兴边富民行动”、“扶持人口较少民族发展”及少数民族特色村落保护与发展工作。

加快建设法治、创新、廉洁和服务型政府。坚持依法行政，把政府工作全面纳入法治轨道，严格按照法定权限和程序行使权力、履行职责。推进政府职能转变，促进政府职能向创造良好发展环境、提供优质公共服务转变。进一步完善政务公开和各领域办事公开制度，加快电子政务系统建设，推进管理和服务方式创新。加强廉政建设，严格落实党风廉政建设责任制，强化审计监督和行政监察，提升行政效能。加大行政问责力度，坚决利用督查问责利器治庸、治懒、治散，解决好不能为、不想为、不敢为的问题。要突出问题导向，坚持底线思维，见贤思齐，敢于担当，确保市委、政府的各项决策部署真正落到实处，以政府的辛苦指数换取群众的幸福指数和满意指数。

各位代表，“十三五”发展的宏伟蓝图已经绘就。让我们紧密团结在以习近平同志为核心的党中央周围，按照自治区党委、政府和市委的总体部署，守望相助，团结奋斗，把祖国北疆这道风景线打造得更加亮丽!
